# Anaesthetic Management of Renal Transplant Surgery in Patients of Dilated Cardiomyopathy with Ejection Fraction Less Than 40%

**DOI:** 10.1155/2014/525969

**Published:** 2014-08-19

**Authors:** Divya Srivastava, Tanmay Tiwari, Sandeep Sahu, Abhilash Chandra, Sanjay Dhiraaj

**Affiliations:** ^1^Department of Anaesthesiology, Mayo Institute of Medical Sciences, Barabanki, Uttar Pradesh, India; ^2^Department of Anaesthesiology, Sanjay Gandhi Postgraduate Institute of Medical Sciences, Lucknow, Uttar Pradesh, India; ^3^Department of Nephrology, Ram Manohar Lohia Institute of Medical Sciences, Lucknow, Uttar Pradesh, India

## Abstract

Cardiovascular disease (CVD) is an important comorbidity of chronic kidney disease, and reducing cardiovascular events in this population is an important goal for the clinicians who care for chronic kidney disease patients. The high risk for CVD in transplant recipients is in part explained by the high prevalence of conventional CVD risk factors (e.g., diabetes, hypertension, and dyslipidemia) in this patient population. Current transplant success allows recipients with previous contraindications to transplant to have access to this procedure with more frequency and safety. Herein we provide a series of eight patients with dilated cardiomyopathy with poor ejection fraction posted for live donor renal transplantation which was successfully performed under regional anesthesia with sedation.

## 1. Introduction 

End stage renal disease (ESRD) [1] is the last stage of chronic kidney disease when glomerular filtration rate is less than 15 mL/min/1.73 m^2^ and renal replacement therapy is essential to sustain life. Renal transplantation is the treatment of choice [1]. Most ESRD patients are anuric or oliguric. Chronically increased intravascular volume may lead to concentric hypertrophy or dilated cardiomyopathy. Dilated cardiomyopathy (DCM) is defined by the presence of (a) fractional myocardial shortening <25% and/or ejection fraction <45% and (b) left ventricular end diastolic diameter >117% excluding any known cause of myocardial disease [2]. Management of patients with severe cardiomyopathies and left ventricular dysfunction is associated with a high morbidity and mortality. LV ejection fraction of ≤35% is considered to be an optimal predictor of postoperative cardiac events [3]. Reduced kidney function is an independent risk factor for adverse postoperative cardiovascular outcomes including myocardial infarction, stroke, and progression of heart failure. An evaluation of 852 subjects undergoing major vascular surgery demonstrated an increase in mortality when serum creatinine was ≥2.0 mg/dL [3]. The patients presented here had dual problems of abnormal physiology due to ESRD and ventricular dysfunction due to DCM, thereby further increasing perioperative cardiovascular risk. 

We describe successful conduction of renal transplant surgery in eight ESRD patients with DCM using combined spinal epidural technique (CSE).

## 2. Cases

Proper preoperative assessment of all eight haemodialysis dependent ESRD patients was done. Relevant details are provided in Table 1.

The patients were informed of their high risk status for anaesthesia. The coagulation profile of all the patients as assessed by bleeding time, prothrombin, and partial thromboplastin time was within normal limits. Informed consent was taken for regional anaesthesia. Immunosuppressants were started two days before. All other medications were continued. Diabetics on hypoglycemics were converted on insulin. Alprazolam 0.25 mg and ranitidine 150 mg were given orally night before operation.

On the day of operation, intra-aortic balloon pump device was kept ready. A five-lead ECG and pulse oximetry were connected to the patient. Oxygenation by face mask was started. Under local anaesthesia, radial artery opposite to site of arteriovenous fistula was cannulated and invasive arterial monitoring started. Right internal jugular vein was also cannulated with a 8.5 F sheath under local anaesthesia. A continuous cardiac output monitoring Swan Ganz Catheter (CCO pulmonary artery (PA) catheter, Edwards Lifesciences) was introduced through the sheath till it got wedged in pulmonary artery. In sitting position and under local anaesthesia an 18 G epidural catheter was placed in T12-L1 interspace. A test dose of 3 mL of 2% xylocaine with adrenaline was given to confirm position of the catheter. A 25 G Quincke's needle was used for injecting 12.5 mg 0.5% hyperbaric bupivacaine and 25 ug fentanyl in L2-L3 intrathecal space. Infusion of 0.25% bupivacaine with 2 ug/mL fentanyl was started via epidural catheter at 3–5 mL/hour. A loading dose of Dexmedetomidine 1 ug/kg was infused over 20 minutes and then titrated at 0.2–0.5 mg/kg/hr to reach a Ramsay sedation score [4] four. 500 mg intravenous methylprednisolone was given at the time of graft anastomosis. In addition serial serum lactate levels were measured.

The mean ± S.D duration of surgery was 3.25 ± 0.45 hours. 2.88 ± 0.353 liters of normal saline was infused intraoperatively. Mean blood loss was 0.483 ± 0.185 liters. Packed red blood cells were used in four patients. Immediate diuresis was observed after reperfusion in all cases. Mean first hour urine output was 0.958 ± 0.160 liters.

**Figure 1:**
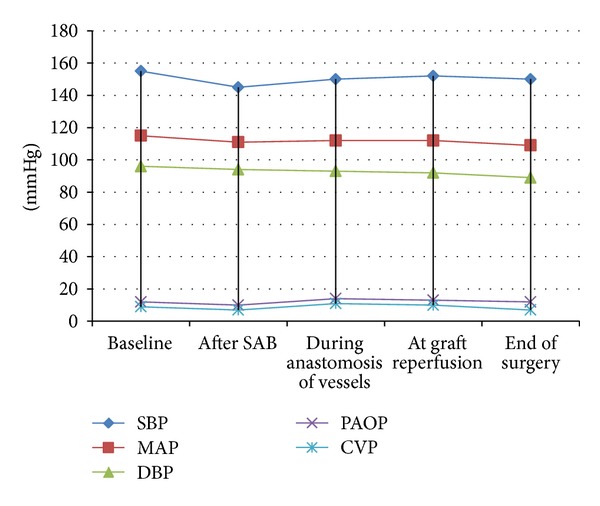
Intraoperative haemodynamics. (Values are mean of readings of eight patients.) SBP: systolic blood pressure, MAP: mean arterial pressure, DBP: diastolic blood pressure, PAOP: pulmonary artery occlusion pressure, CVP: central venous pressure, and SAB: subarachnoid block.

**Figure 2:**
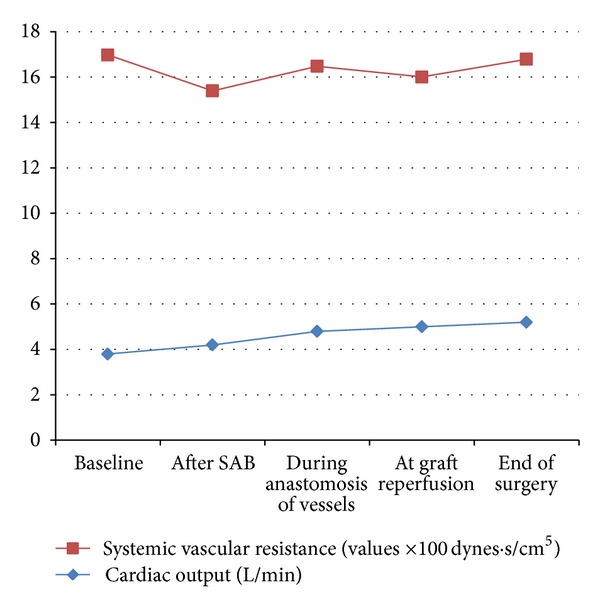
Intraoperative haemodynamics. (Values are mean of readings of eight patients.) SAB: subarachnoid block.

Vigilance monitor, Edward Lifesciences, USA, was used to monitor cardiac output (CO) and systemic vascular resistance (SVR). The target was to keep CO above 4 L/min and SVR between 800 and 1200 dynes · sec/cm^5^. Noradrenaline infusion was started at 2 ug/min and titrated to maintain SVR and mean arterial pressure. Dobutamine infusion was started at 5 ug/kg/min and titrated to maintain CO.  Figure 1 shows mean intraoperative parameters and Figure 2 shows mean variations in systemic vascular resistance throughout the procedure and continuous increase in the mean cardiac output in response to anaesthetic management. Dobutamine infusion was used in three patients (37.5%). Noradrenaline was required in two patients (25%). Infusions were tapered off over 3–6 hours after surgery. 0.125% bupivacaine with 2 ug/mL fentanyl solution was used for postoperative patient controlled epidural analgesia.

## 3. Discussion

The present case series describes successful anaesthetic management of patients having DCM and ESRD.

Preoperative assessment [2] is important and the following factors must be taken care of.

- Patients of DCM are on the verge of developing cardiac failure and patients of ESRD oscillate between hypovolemia and hypervolemia, so preoperative volume assessment is very important.
- Arrhythmias like ventricular tachyarrhythmias and atrial fibrillation are commonly associated with DCM [5]. The risk is increased in dialysis dependent patients because of dyselectrolytemia. Preoperative assessment and correction of electrolytes particularly are therefore essential.
- Anemia is a frequent finding in ESRD patients. Haemoglobin optimization is required for maintaining adequate oxygenation of the blood.
- Oxygen carrying capacity is also dependent on cardiac output (CO). To improve cardiac output inotropes may be utilized. In the event of failure of inotropes, biventricular synchronized pacing or an intra-aortic balloon pump may be required.

*Intraoperative Monitoring*. Pulmonary artery catheterization and invasive arterial pressure monitoring are recommended in patients with left ventricular dysfunction undergoing renal transplantation [1].

The goals of anaesthetic management are [1, 2]

- maintenance of myocardial contractility,
- avoiding drug induced myocardial depression,
- prevention of increase in afterload,
- maintenance of adequate preload while preventing fluid overload,
- avoiding tachycardia,
- maintenance of adequate mean arterial pressure for renal perfusion,
- avoidance of nephrotoxic drugs.

Kidney transplantation is usually performed under general anaesthesia (GA) with endotracheal intubation and mechanical ventilation [1]. GA provides adequate muscle relaxation and depth required for the surgery. However myocardial depressant effects of anaesthetic agents and stress of laryngoscopy and intubation are not desirable in these patients. Patients of left ventricular dysfunction have increased incidence of deep vein thrombosis (DVT) and pulmonary embolism [2]. Keeping the above facts in mind combined spinal epidural (CSE) technique was adopted with low dose spinal block and extension with epidural catheter. Renal transplant surgeries have previously been conducted successfully under CSE [6].

Regional anaesthesia was chosen over GA because it attenuates neurohumoral stress response to surgery, produces vasodilatation thereby decreasing afterload, decreases incidence of DVT, pulmonary embolism, and respiratory depression, avoids polypharmacy, decreases transfusion requirements, and is associated with early recovery [7]. Epidural anaesthesia causes moderate fall in SVR and helps to maintain forward left ventricular flow in systolic dysfunction. It also provides an effective means of postoperative analgesia in a situation where nonsteroidal anti-inflammatory drugs are contraindicated and excessive intravenous opioids may cause respiratory depression. Good postoperative pain management avoids increase in SVR and heart rate and maintains postoperative cardiovascular stability. One of the concerns with use of regional anaesthesia in ESRD patients is their abnormal platelet function and use of heparin during dialysis. Platelet functions and coagulation profile were within normal limits in our patients and strictly heparin free haemodialysis was performed 24 hours before surgery.

CSE was preferred over continuous epidural technique, because spinal anaesthesia provides a reliable dense block and muscle relaxation to begin with and the duration of anaesthesia can be extended with epidural catheter. A low dose of bupivacaine heavy was given as low dose bupivacaine plus fentanyl has been shown to provide stable haemodynamics with reduced requirement of vasoconstrictive drugs or fluids [8]. Sanatkar et al. [9] observed that decrease of blood pressure after a low dose of subarachnoid block in patients with low ejection fraction was lower than in patients with EF > 40%. They attributed the finding to increase of cardiac output due to reduction of systemic vascular resistance and afterload in patients with low ejection fraction more than control group.

Dexmedetomidine, a *α*2adrenoceptor agonist [10], infusion was used for anxiolysis and sedation. At high doses hypotension and bradycardia have been observed, but no such side effects were noted, probably because of the low dose used. Dexmedetomidine was selected because it provides effective sedation without respiratory depression and it has sympatholytic, cardioprotective, and renoprotective actions [10]. It has also been used for its antiarrhythmic properties before [11, 12] and was of help to keep a check on tachycardia.

The overall anesthetic goal for the newly transplanted kidney is to maintain intravascular volume and avoid decreased perfusion by maintaining MAP. Adequate volume expansion and a CVP of 10−15 mmHG are required [1]. However excessive hydration may lead to congestive cardiac failure in patients with poor left ventricular function [2]. So our fluid therapy was guided by cardiac filling pressures. Pulmonary artery occlusion pressures were maintained between 11 and 14 mmhg.

Hypotension should be avoided to prevent both myocardial and renal allograft hypoperfusion. To counteract the peripheral vasodilating effects of anaesthesia norepinephrine infusion was used to maintain adequate blood pressure [2]. Inotropes like dobutamine are used to increase cardiac output to maintain adequate tissue perfusion. Serial lactate values within normal limits proved adequacy of peripheral perfusion [13].

The postoperative period remained uneventful. All patients were discharged 15 to 20 days later with normal graft functions on oral immunosuppressants, antihypertensives, and oral hypoglycemics as required.

The perioperative cardiovascular complication rate in renal transplant surgeries is 6% [1]. DCM in itself has poor prognosis with only 25–40% survival at end of five years [14, 15]. The predictors of poor prognosis are an ejection fraction of less than 25%, left ventricular end diastolic dilatation, a hypokinetic left ventricle, and presence of mitral and tricuspid regurgitation [14]. Four of our patients (50%) had ejection fraction less than 25%. Incidence of global hypokinesia was 50% (4/8). Mitral regurgitation was seen in seven patients (87%) and three of these also had tricuspid regurgitation. All the four bad prognostic factors were present in two patients (25%). The common causes of death in these patients are cardiac failure and malignant arrhythmias [2]. Reports of successful conduction of surgeries under both general and regional anaesthesia in DCMP patients with systolic dysfunction are present but to the best of our knowledge there is no data available on conduction of renal transplant surgery in these compromised patients.

Anesthetic management of a patient having dual problems of ESRD and DCMP is a challenge for the anaesthesiologist. The perioperative management of above patients teaches us that meticulous preoperative optimization, close intraoperative monitoring, tailoring the anaesthetic plan to patient's specific needs, optimization of fluid status and haemodynamics, and watchful postoperative care are the key to a successful renal transplant in these high risk patients.

## Figures and Tables

**Table 1:** Important patient information.

	Age (years)/sex	Comorbidities	Transthoracic echocardiography	Left ventricular EF (%)	Oral medications
1	30/M	Htn,	Dilated LV, severe MR, mild TR, mild PAH	24	Metoprolol, OHA
2	34/M	Htn, type II DM	Concentric LVH, dilated LV, global hypokinesia, no PAH	35	Clonidine, nifedipine, OHA
3	38/M	Htn, type II DM	Mild concentric LVH, dilated LV, mild MR, no PAH	40	Nifedipine, clonidine, metoprolol, prazosin, insulin
4	38/F	Htn	Moderate LVH, dilated LV, global hypokinesia, mild MR, no PAH	24	Metoprolol, clonidine
5	42/M	Htn, type II DM	Mild concentric LVH, dilated LV, mild MR, mild PAH	34	Metoprolol, insulin
6	45/M	Htn	Mild concentric LVH, dilated LV global hypokinesia severe TR, moderate MR, moderate PAH	15	Amlodipine, clonidine, metoprolol
7	46/M	Htn, type II DM	LVH, dilated LV, mild MR, moderate PAH	40	Amlodipine, metoprolol, prazosin, insulin
8	46/M	Htn	Dilated LV, global hypokinesia, moderate MR, moderate TR, mild PAH	25	Metoprolol

M: male, F: female, Htn: hypertension, DM: diabetes mellitus, LV: left ventricle, MR: mitral regurgitation, TR: tricuspid regurgitation, PAH: pulmonary artery hypertension, OHA: oral hypoglycaemic agents, LVH: left ventricular hypertrophy.
